# The nature of science: The fundamental role of natural history in ecology, evolution, conservation, and education

**DOI:** 10.1002/ece3.10621

**Published:** 2023-10-23

**Authors:** Karma Nanglu, Danielle de Carle, Thomas M. Cullen, Erika B. Anderson, Suchinta Arif, Rowshyra A. Castañeda, Lucy M. Chang, Rafael Eiji Iwama, Erica Fellin, Regine Claire Manglicmot, Melanie D. Massey, Viviana Astudillo‐Clavijo

**Affiliations:** ^1^ Museum of Comparative Zoology and Department of Organismic and Evolutionary Biology Harvard University Cambridge Massachusetts USA; ^2^ Department of Ecology and Evolutionary Biology University of Toronto Toronto Ontario Canada; ^3^ Department of Invertebrate Zoology Royal Ontario Museum Toronto Ontario Canada; ^4^ Department of Geosciences Auburn University Auburn Alabama USA; ^5^ Negaunee Integrative Research Center Field Museum of Natural History Chicago Illinois USA; ^6^ The Hunterian University of Glasgow Glasgow UK; ^7^ Department of Earth and Space Royal Ontario Museum Toronto Ontario Canada; ^8^ Department of Biology Dalhousie University Halifax Nova Scotia Canada; ^9^ Ecosystems and Ocean Sciences Pacific Region, Fisheries and Oceans Canada Sidney British Columbia Canada; ^10^ California Science Center Los Angeles California USA; ^11^ Departamento de Genética e Biologia Evolutiva, Instituto de Biociências Universidade de São Paulo São Paulo Brazil; ^12^ Department of Biology McGill University Montreal Quebec Canada; ^13^ Department of Zoology The University of British Columbia Vancouver British Columbia Canada; ^14^ Ecology and Evolutionary Biology University of Michigan Ann Arbor Michigan USA

**Keywords:** biodiversity, conservation biology, earth sciences, equity, museums, policy, science education, taxonomy, undergraduate education

## Abstract

There is a contemporary trend in many major research institutions to de‐emphasize the importance of natural history education in favor of theoretical, laboratory, or simulation‐based research programs. This may take the form of removing biodiversity and field courses from the curriculum and the sometimes subtle maligning of natural history research as a “lesser” branch of science. Additional threats include massive funding cuts to natural history museums and the maintenance of their collections, the extirpation of taxonomists across disciplines, and a critical under‐appreciation of the role that natural history data (and other forms of observational data, including Indigenous knowledge) play in the scientific process. In this paper, we demonstrate that natural history knowledge is integral to any competitive science program through a comprehensive review of the ways in which they continue to shape modern theory and the public perception of science. We do so by reviewing how natural history research has guided the disciplines of ecology, evolution, and conservation and how natural history data are crucial for effective education programs and public policy. We underscore these insights with contemporary case studies, including: how understanding the dynamics of evolutionary radiation relies on natural history data; methods for extracting novel data from museum specimens; insights provided by multi‐decade natural history programs; and how natural history is the most logical venue for creating an informed and scientifically literate society. We conclude with recommendations aimed at students, university faculty, and administrators for integrating and supporting natural history in their mandates. Fundamentally, we are all interested in understanding the natural world, but we can often fall into the habit of abstracting our research away from its natural contexts and complexities. Doing so risks losing sight of entire vistas of new questions and insights in favor of an over‐emphasis on simulated or overly controlled studies.

## Graphical abstract

Natural history research is crucial for any competitive science curriculum, but it faces a wide array of challenges in the current academic environment, including a critical lack of funding and support. In this paper, we review the critical importance of natural history science for studying evolution, ecology, conservation, the earth science, as well as its role in creating a scientifically literate society.

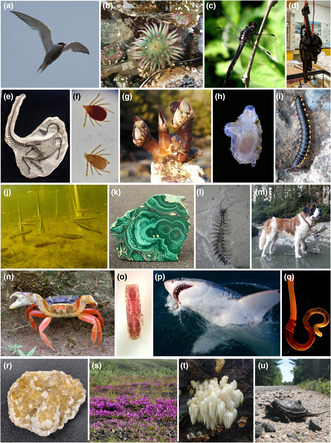

## 1 INTRODUCTION

**FIGURE 1:**
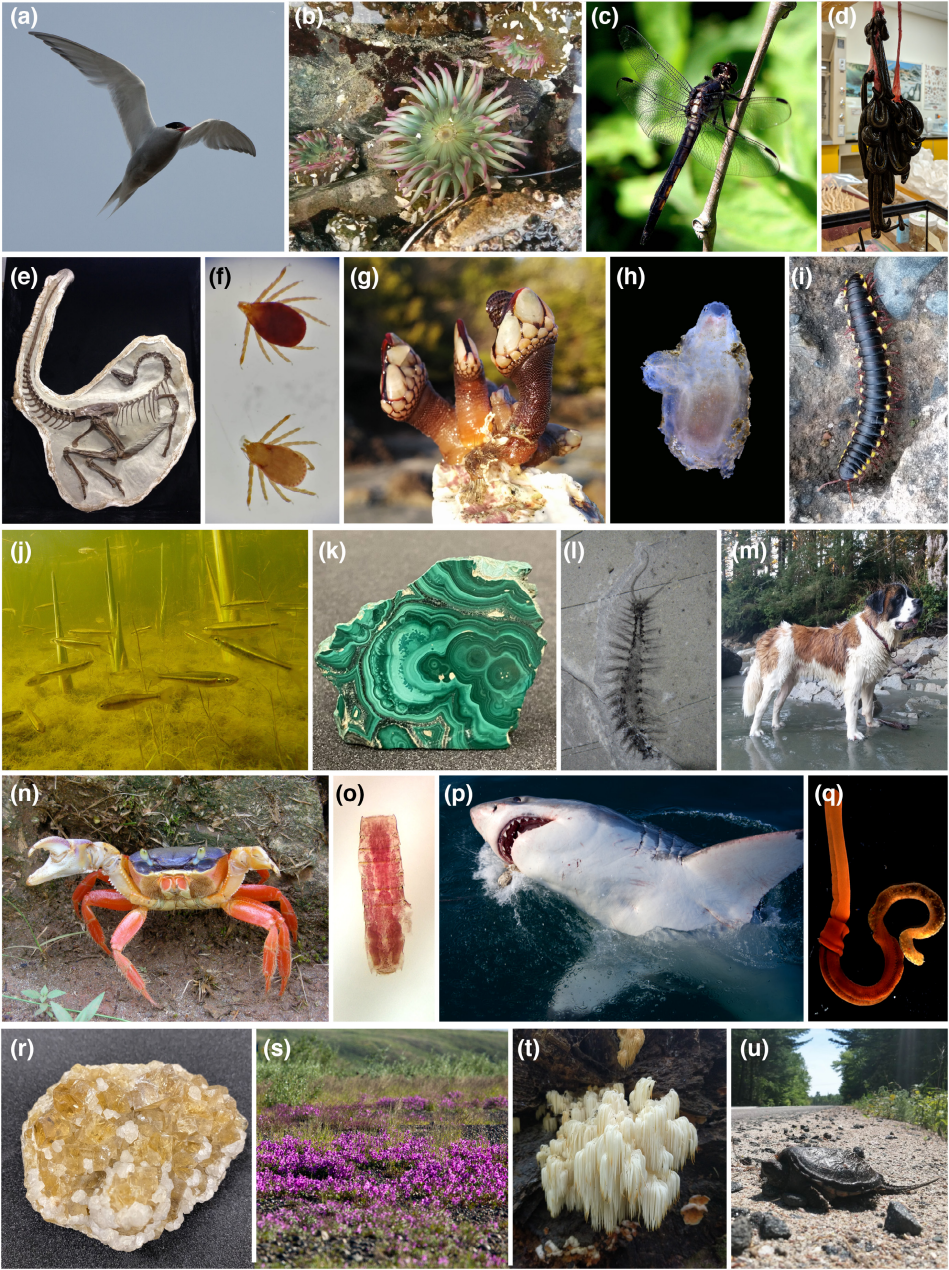
A selection of specimens representing a small part of the diversity covered by natural history research, with a focus on specimens studied by the co‐authors. (a) *Sterna paradisaea*. (b) *Anthopleura* sp. (c) *Aeshna canadensis*. (d) *Hirudo verbana*. (e) *Ornithomimus edmonticus*. (f) *Ixodes scapularis* and *Derma centorvariabilis*. (g) *Pollicipes* sp. (h) *Ascidiella* sp. (i) *Asiomorpha coarctata*. (j) *Clinostomus elongatus*. (k) Malachite (Hunterian Museum M6752). (l) *Kootenayscolex barbarensis*. (m) *Canis lupus familiaris*. (n) *Cardisoma crassum*. (o) Unidentified Kinorhyncha. (p) *Carcharodon carcharias*. (q) *Saccoglossus pusillus*. (r) Fluorite with calcite (Hunterian Museum M623). (s) *Saxifraga oppositifolia*. (t) *Hericium erinaceus*. (u) *Chelydra serpentina*. Photo credits: a, c, p, s: Thomas M. Cullen. b, d, g, i, m, t: Danielle de Carle. e: David C. Evans. f, u: Erica Fellin. h, o, q: Karma Nanglu. j: Rowshyra Castañeda. k, r: Erika B. Anderson, The Hunterian, University of Glasgow. l: Jean‐Bernard Caron. n: Javier Luque.

Natural history can be broadly defined as the search for and description of patterns through direct observation of the natural world. This encompasses organisms, physical materials, and environments, as well as the processes that govern and generate them (Figure 1). Natural history research may be exploratory or involve direct hypothesis testing. In either case, it relies primarily on observational data collected directly from those sources as they exist in situ, without experimental manipulation. Many branches of science—including ecology, evolutionary biology, geology, taxonomy and systematics, paleontology, and conservation biology—have their roots in natural history. Today, natural history data and research continue to be a quintessential complement to theoretical and experimental methods of inquiry: they provide the baseline knowledge of natural systems required to extrapolate broader theory, test hypotheses, and uncover the general principles that govern natural systems.

This definition highlights why the study of natural history serves as an organic introduction to the biological and earth sciences: the simple act of observing the natural world and formulating questions about what is observed, even without an appreciation for the intellectual legacy of the discipline, has already set the curious down the road to becoming informal natural historians. In this way, although natural spaces are not equally accessible across all communities (Rowland‐Shea et al., 2020; Shanahan et al., 2014), the barrier to practicing the foundations of this science is overcome by a trip to a local public park, backyard, or beach.

“Biophilia,” an intrinsic affinity with the natural world and all living things (Ulrich, 1993), is an essential part of the human experience. This interest is not a recent development: an appreciation for natural ecosystems and one's place in them is a significant feature of cultural and spiritual practices throughout history, with veneration and stewardship of nature being a common thread (Egri, 1997). Likewise, one cannot overlook the many communities that rely on the responsible use of natural resources for economic purposes and sustenance. It is unsurprising, then, that the study of natural phenomena—and the biosphere in particular—has been, and continues to be, a cornerstone of the public consciousness, whether that is in the form of public education venues such as museums and science centers, national parks, eco‐tourism, or art and other forms of popular media.

Its approachability, ubiquity, and inherent relevance all help to explain why natural history is, in a colloquial sense, broadly familiar to the public at large (Ballard et al., 2017). However, we argue that these very strengths lead to a serious undervaluation of natural history as a field of research with broad ramifications. The critical question, then, is: Why is the study of natural history overlooked or, in some cases, outright maligned within academic circles? We argue that a central factor is the lack of appreciation for the overarching potential of natural history data and practices to illuminate principles of major scientific and social significance. In other words, there is insufficient understanding of the actual process of conducting natural history research, including the principle questions asked, the methods employed, the underlying motivations, and its relevance, not only to science but to society as a whole. This is a particularly glaring omission at a time when harmful social and ecological trends could be ameliorated with the foundational skillset conferred by a natural history education.

**FIGURE 2:**
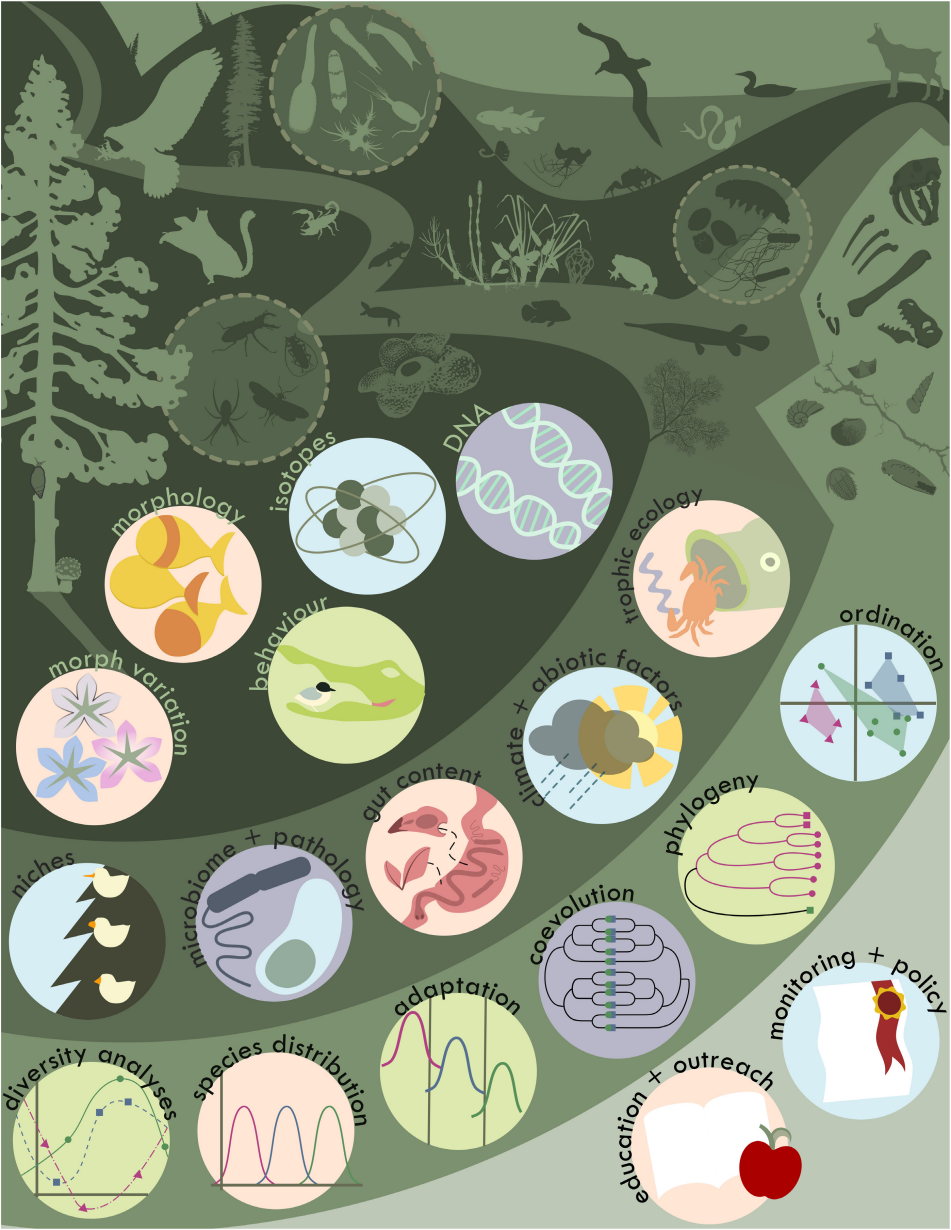
Natural environments (top of the figure) provide not just the inspiration that begins the scientific process but also the data needed to generate and test hypotheses and ultimately understand the world. The concentric rings of icons depict how specimens such as plants, animals, and minerals underlie all aspects of research. Data, such as the morphology of animals and the structure of minerals (first ring), provide the means to understand fundamental features of the natural world, such as how niches are partitioned or how abiotic factors influence community structure (second ring). In turn, knowledge from the second ring provides the substrate for the analyses (third ring), often associated with the words “science” or “research.” The results of these analyses can not only inform future studies that return to natural systems for validation and further study but also influence societies at large through education initiatives and by informing policy decisions (fourth ring).

Here, we summarize the threats facing natural history at all levels. Then, to highlight the fundamental role of natural history in ecology, evolution, conservation, and education, we bring forth examples of the range of research made possible by natural history data. These examples run the entire gamut of major topics in ecology, evolutionary biology, and earth sciences, including species diversification, trait evolution, community ecology, trophic dynamics, environmental and climatic reconstruction, and conservation biology (Figure 2). Using a series of case studies, we synthesize the relevance of natural history, with particular emphasis on the pitfalls encountered when natural history data are ignored. We also discuss the broader impacts of a natural history education on academia and society at large. Finally, we outline ways to support natural history research and education, and integrate natural history data with experimental and simulation‐based research (Box 1) to produce more robust and nuanced results.

## 2 NATURAL HISTORY: A DISCIPLINE IN DANGER

Natural history research and education face threats at many levels, from university departments, to institutions, to governments. At the level of university and college departments, pedagogical systems that facilitate knowledge transfer to natural history trainees are facing devaluation: departmental support for natural history courses is declining at many highly ranked post‐secondary institutions, while bias against organismal biology increases (Gropp, 2004). Our own experiences echo these trends: frequently, we have been told that natural history is “less rigorous” than controlled laboratory or experimental science. This unfortunate sentiment can lead to a lack of natural history experts among department faculty, which in turn leads to a natural history‐depauperate undergraduate curriculum (Bradley et al., 2014; Ernst et al., 2014). Presently, many ecology and evolutionary biology departments are moving toward highly theoretical or simulation‐based research programs that are increasingly removed from the complex and often comparatively poorly documented reality of natural systems (Ernst et al., 2014; Tewksbury et al., 2014). The issue is compounded by the fact that many early‐career scientists feel inadequately trained to teach natural history courses (Barrows et al., 2016). Taxonomy as a discipline has been hit particularly hard: decades of specialist knowledge and irreplaceable familiarity with study systems are being lost before they can be transferred to new generations of researchers.

Some nations have pledged to improve the status of taxonomic research; Australia, for example, has proposed to document the remaining species within the country. This endeavor has a projected cost of $824 million, yet has potential benefits valued at $3.7–28.9 billion through impacts on biosecurity, agriculture, biodiscovery, and biodiversity (Deloitte, 2020). However, these types of initiatives seem to be the exception: funding cuts like those seen in countries such as Brazil and South Africa (Herbert, 2001; Santos & Carbayo, 2021; and see below) seem more common.

Worldwide, there have been numerous budget cuts to natural history institutions over the past several decades (Coniff, 2016; Dalton, 2003; Kemp, 2015; Overbeck et al., 2018). Natural history museums routinely face existential crises from underfunding, redirection of funding, staff shortages, and disrepair (Andreone et al., 2014; Britz et al., 2020; Liston, 2011; Rohwer et al., 2022; Shen, 2012), despite serving critical roles as repositories that document what exists and has existed on the planet and how that diversity has changed over macroecological, macroevolutionary, and geological timescales. Practices that form the foundations of natural history—including species description, observational records, systematics, and biodiversity surveys—are underfunded and under threat of total disbandment. All of this is occurring within the social context of manifold ecological crises, such as climate change, habitat destruction, zoonotic disease outbreaks, and the overexploitation of natural resources, whose full complexity cannot be grasped without a thorough understanding of the natural systems in which they exist (Bakker et al., 2020; Britz et al., 2020).

### 2.1 Case study—The state of natural history support in Brazil, a global biodiversity hotspot

The Brazilian territory is composed of a wide range of biomes that house a considerable part of the global biodiversity. The estimated total number of species inhabiting this territory is 1.8 million (13.1% of estimated global diversity), with only 170,000 species formally described (representing 9.5% of the global number of described species) (Lewinsohn & Prado, 2005). Some Brazilian biomes, such as the Amazon and the Atlantic forest, receive great international attention due to their high level of diversity and endemism, but also due to increasing pressure on these areas driven by political and economic factors. The Atlantic forest, for example, is home to more than 500 amphibian species (Vancine et al., 2018), 200 species of mammals (Bovendorp et al., 2017; Souza et al., 2019), and 745 species of birds (Hasui et al., 2018). It currently faces major challenges to conservation, with only 28% of its original vegetation remaining (Rezende et al., 2018). Furthermore, the Brazilian territory also houses the majority of Indigenous territories in South America, which show low rates of deforestation and are under high levels of protection. Therefore, these areas are of great importance for both cultural and species conservation within the continent and globally.

With all its diversity, it is natural to assume that biodiversity and conservation research would be a priority in Brazil. And in fact, from 2004 to 2013, there was a major increase in general research funding that resulted in a steady increase in indicators of research quality (12.7% and 18.3% in the number of research papers and citations, respectively) (Fernandes et al., 2017). Biodiversity studies also greatly benefited during this period, with an increase in the number of graduate programs in ecology, zoology, and botany. But since then, science and technology have suffered from sequential budget cuts that threaten Brazilian science and science education (Gibney, 2015; Moura & Camargo Junior, 2017).

The year 2016 saw major steps backward in science and education. The federal budget for science, technology, education, and other key areas was frozen for 20 years, bringing funding to levels comparable only to values from before 2004. These major cuts impacted science rapidly (Escobar, 2018). Brazilian researchers started to flee to other countries, and the number of scientific papers declined in the years that followed (Angelo, 2017; Oliveira et al., 2020). Graduate programs were not immune to these major budget cuts. In many cases, the continuity of postdoctoral, graduate, and undergraduate research fellowships was threatened, impacting roughly 80,000 fellowships (Andrade, 2019; Petherick, 2017). In the field of natural history, the situation is similar. The number of fellowships awarded to fields of natural science, such as taxonomy, morphology, and paleontology, has declined considerably since 2014, even within the Fundação de Amparo à Pesquisa do Estado de São Paulo (FAPESP), a major funding agency in Brazil (https://bv.fapesp.br/pt/540/bolsas/, FAPESP, n.d.‐a; accessed on August 23, 2023).

Research labs and natural history collections have also encountered major funding problems. In 2018, a major fire destroyed the National Museum in Rio de Janeiro, the biggest natural history museum in the country. Among the specimens deposited at the museum at that time were more than 40,000 Indigenous and African artifacts; over 5 million insect specimens; an 11,500‐year‐old skull (Luzia, the oldest human remains in the Americas); in addition to thousands of specimens of fossils, meteorites, and other natural history collections (Araujo, 2019; Escobar, 2018; Zamudio et al., 2018) that were portraits of the evolution of culture and biodiversity in South America. Initially, 90% of the collections were estimated to be lost, interrupting the majority of the activities performed in the museum, such as curatorial work and research. Later, several items were recovered from the ashes, but the damage to current and future natural history research was already done (Mega, 2019). The tragedy, however, was not a surprise. The lack of building maintenance resulting from the major budget cuts in the years that preceded the fire was known, and the tragedy was foretold (Escobar, 2018).

Unfortunately, the fire that devastated the National Museum was not an isolated case. Years before, another fire destroyed over 80,000 specimens of snakes and more than 450,000 specimens of spiders and scorpions deposited at Instituto Butantan. The full legacy of this destruction has yet to be defined, since Butantan is a major center for toxins, vaccines, and antivenom research (Kmech, 2010).

In addition to decreasing numbers of research fellowships and inadequate maintenance of natural history collections facilities, the amount of funding awarded to principal investigators also declined after 2014 (https://bv.fapesp.br/pt/6/auxilios‐regulares/, FAPESP, n.d.‐b; accessed on August 23, 2023). Without major funding and available scholarships, Brazilian natural history research has been struggling. Although other national collections have a good representation of the territory's biodiversity, many important specimens that were originally collected in Brazil are not deposited in local museums. Instead, due to the legacy of colonization, many taxonomically important specimens are deposited abroad, increasing the cost of basic taxonomic and other collection‐based research projects by necessitating travel to multiple international natural history museums and other collections (Pires‐O'Brien, 1993). Although the colonization ended more than 200 years ago, the problem persists, with specimens being illegally collected and sent to research institutions abroad without the involvement of local scientists or collections and shipment permits. A classic example is *Ubirajara jubatus*, considered to be the first non‐avian dinosaur found in South America to possess structures that are hypothesized to be precursors of feathers. It was suggested that this specimen was illegally collected and deposited in Germany (Cisneros et al., 2022; Lenharo & Rodrigues, 2022). After years of campaigning against colonization in science, the specimen returned to Brazil in 2023 (Rodrigues, 2023).

Underfunding also impacts access to cutting‐edge technology that is becoming increasingly popular in natural history studies. Studies involving any sort of high‐throughput sequencing, for example, are becoming more common and accessible to researchers in the global north due to decreases in sequencing costs. However, these large‐scale sequencing projects are still relatively uncommon in Brazil, where a lack of available funding means these analyses are still cost‐prohibitive. Striking evidence can be found among papers included in the Web of Science database that contain the search term “phylogenomics” (https://www.webofscience.com/, accessed on August 23, 2023). When filtered by country, research labs from the USA and China have produced 2,015 and 943 studies, respectively, between 2020 and 2023. In the same period, research labs from Brazil published only 288 phylogenomic studies. This huge discrepancy highlights the extreme differences in access to cutting‐edge technologies among natural history researchers and labs in these three countries.

## 3 NATURAL HISTORY AND EVOLUTION

The field of evolutionary biology aims to understand the diversity and diversification of organisms, both extant and extinct, at various levels of organization (e.g., from genes to phyla). Some of the major questions addressed by evolutionary biologists include: How are organisms related? How do different mechanisms (e.g., stochastic, geographical, adaptive, biotic, and abiotic) drive lineage and phenotypic diversification? How do microevolutionary processes occurring at the population level translate to macroevolutionary patterns? Why is diversity not evenly distributed across organismal groups (e.g., paleognaths vs. passerine birds) or environments (e.g., temperate vs. tropical)? How are humans affecting the diversity and evolution of organisms?

Empirical work, often conducted with model organisms in a controlled laboratory setting, has been indispensable for understanding evolutionary processes. Decades of research on *Drosophila* (Bellen et al., 2010), *Arabidopsis* (Provart et al., 2016), and *Caenorhabditis* (Nigon & Félix, 2017) have served to elucidate principles of inheritance, adaptation, and speciation. However, beyond the walls of a laboratory and the control of designed experiments lie the natural environments from which researchers draw initial inspiration and hypotheses and in which theories are truly put to the test. Here, we highlight some of the ways in which natural history is a crucial component of evolutionary research and present a case study that draws on some of these contributions.

Perhaps first and foremost, one simply cannot begin to study organismal evolution if they are unaware of the organisms themselves. Elaborate theoretical models describing fundamental evolutionary processes, such as population dynamics, inheritance, adaptation, speciation, and extinction, all have their origins in observing and taking note of taxa. For example, it is impossible to study speciation and extinction without first finding, describing, and classifying species—monumental tasks, given the vast diversity of life on earth that befalls the often underappreciated field of taxonomy. Beyond providing an inventory of organisms, phylogenetics and taxonomic classification provide valuable hypothesis‐generating information and statistical controls (Baron et al., 2017; Felsenstein, 1985; Jékely et al., 2015; Lehmberg et al., 2018) for many lines of evolutionary research. For example, studying evolutionary convergence relies on an understanding of how putatively convergent taxa are related and to what extent they represent distinct lineages (Stayton, 2015).

Natural historians are also instrumental in assessing the applicability of evolutionary principles to the real world. Evolutionary processes investigated through laboratory or common garden experiments are often, by necessity, limited in their temporal and spatial scope. However, the time and geographical space over which they occur in real life can be vast, during which the intricacies of nature, like the presence of other organisms (e.g., predators and competitors) and unforeseen occurrences (e.g., major climatic events), can affect the process in unexpected ways. As a result, while one might develop a thorough understanding of how a particular evolutionary process, such as adaptation, operates, it remains difficult to predict its outcome or assess its real‐world significance within the confines of a lab. For example, whereas one might expect known selective pressures to drive trait evolution in one direction, unaccounted conflicting functional demands, many‐to‐one mapping of form and function, and behavioral adaptation may instead produce distinct phenotypes that remain unexplained without additional natural history information (Moran et al., 2018; Wainwright et al., 2005). Only by embracing the messiness of nature through complementary natural history research, such as field experiments (Endler, 1990; Losos et al., 1997) or inference from museum specimens (Astudillo‐Clavijo et al., 2015; Burns & Sidlauskas, 2019; Maestri et al., 2017), can a more holistic evaluation of fundamental evolutionary processes emerge.

As understanding of major evolutionary principles continues to progress, it is important to remember the ultimate purpose of evolutionary biology: to elucidate the origins of life on Earth and the processes and mechanisms that generate biological diversity. As such, researchers must strive to keep that very life—be it animals, plants, or microorganisms—front of mind so that their research remains firmly rooted in and relevant to the true products of the processes they seek to understand.

### 3.1 Case study—The ecology of continental radiations: using museum specimens to elucidate historical ecological patterns and the role of adaptation in driving continental diversification

Investigating the drivers of continental diversity is an area of evolutionary research that has relied heavily on natural history knowledge and collections (Arbour et al., 2019; Burns & Sidlauskas, 2019; Claramunt, 2010; López‐Fernández et al., 2013; Maestri et al., 2017). Ecological opportunity leading to adaptive radiation is considered an important source of species and phenotypic diversity (Schluter, 2001; Simpson, 1944; Stroud & Losos, 2016). Studying the influence of ecological adaptation on mainland diversity has remained difficult due to the challenges of quantifying taxonomic diversity and amassing ecological data for species‐rich clades distributed over very broad spatial scales. Nonetheless, macroevolutionary research at continental scales is possible with data sourced from museum collections and informed by decades of natural history research.

While there are many creative ways to study ecological adaptation at macroevolutionary scales, here we highlight a few major “natural history building blocks” common to various studies: (1) species description and diversity estimates; (2) phylogenetic hypotheses; and (3) functional morphology. These basic natural history sourced building blocks have served to infer the ecology of mainland diversification across several groups of organisms, environments, and timescales.

The work of taxonomists is essential from the very beginning. First, diversity cannot be reliably quantified without the discovery and delineation of species. Such information is available for many continental taxa (e.g., fishes, birds, mammals, etc.) thanks to the unending cycle of collecting specimens in the field, cataloging specimens (e.g., whole specimens, tissue samples, etc.) in museums and other collections, classifying and describing species, and returning to resample or sample new field sites. This process is necessarily laborious and requires a keen familiarity with the organisms. The end result is a collection of species accounts that provides invaluable information about their distribution, ecology, and behavior. Unfortunately, taxonomic experts are somewhat of an endangered species themselves (discussed in more detail elsewhere, e.g., Engel et al., 2021) at a time when several species‐rich groups (e.g., invertebrates and fishes) and environments (e.g., the Amazon) remain gravely under‐sampled or poorly delineated while also under threat of massive biodiversity loss (Joppa et al., 2011; Myers et al., 2000; Smiley et al., 2020).

Once a sizable sample of taxa has been collected, researchers can use morphological and/or molecular data, available through museum specimens, to infer phylogenetic hypotheses. A robust phylogeny is a vital component of macroevolutionary research, not just as a statistical control (Felsenstein, 1985), but also for refining taxonomic classification schemes, reconstructing historical events (e.g., ecological or geographic shifts), and informing hypotheses for comparative evolutionary analyses (Baron et al., 2017; Hulsey et al., 2010; Jékely et al., 2015; McGee et al., 2020; Oliveros et al., 2019; Upham et al., 2019).

With a phylogeny and informed hypotheses in hand, one can begin inferring past ecological patterns. This is truly where multiple lines of natural history work intersect. Valuable ecological information can be attained for large and widely distributed clades by measuring ecologically relevant traits (i.e., functional morphology) on preserved specimens in natural history collections (e.g., Arbour et al., 2019; Astudillo‐Clavijo et al., 2015; Claramunt, 2010). But which traits should be measured? A long history of research looking into the relationship between physical form and ecological function in specific taxonomic groups can be used to select traits that are expected to vary with ecology (e.g., Webb, 1984). Measurement of these traits on museum specimens can then be mapped onto a phylogeny comprising the same taxonomic representation using comparative phylogenetic approaches to answer specific questions about the clade's evolutionary history.

Knowledge of a clade's natural history makes it possible to focus analyses on only the most relevant questions and hypotheses of diversification. That is, it allows researchers to primarily consider scenarios that make biological sense. Familiarity with historical biogeography, the discovery and description of fossils, and phylogenetic relationships have all contributed to the identification of continental taxa whose origins coincide with evolutionary events that may have created ecological opportunities, like the extinction of competitors or the creation of novel habitats. For example, following the break‐up of Gondwana, South America saw the extinction of several aquatic lineages with repeated marine incursions and the creation of novel habitats with the recession of marine waters and Andean uplift (López‐Fernández & Albert, 2011). For subclades thought to have benefited from such biogeographic events, researchers have looked for shifts to higher rates, or early bursts, of functional morphological evolution (Astudillo‐Clavijo et al., 2015; Burns & Sidlauskas, 2019; Burress et al., 2018; López‐Fernández et al., 2013). Early bursts are considered a trademark of adaptive evolution in response to ecological opportunity (Gavrilets & Losos, 2009; Simpson, 1944; Stroud & Losos, 2016). More precise detail regarding the nature of ecological adaptation during diversification has been inferred by fitting evolutionary models to functional morphological data and phylogeny. The fit of alternative models that incorporate shifts to novel adaptive optima in specific lineages (e.g., Ornstein–Uhlenbeck models), akin to Simpson's adaptive landscape (Simpson, 1944), are often tested against each other and against a null model that predicts random non‐directional evolutionary change (Arbour et al., 2019; Astudillo‐Clavijo et al., 2015; Burns & Sidlauskas, 2019; Butler & King, 2004; Ingram & Mahler, 2013; Khabbazian et al., 2016; López‐Fernández et al., 2013). Given the functional nature of selected traits, shifts to new optima could be interpreted as evolutionary transitions in suites of traits that optimize performance under different ecological circumstances, which in some cases may be concentrated early in a clade's history, consistent with the expectations of ecological opportunity (Astudillo‐Clavijo et al., 2015). The phylogenetic position of these shifts should be informed by a clade's natural history. For example, alternative models under consideration have been based on interspecific variations in diets or geographic regions and environmental distributions (Arbour et al., 2019; Arbour & López‐Fernández, 2016; Burns & Sidlauskas, 2019; Rossoni et al., 2019). Natural history knowledge enhances comparative phylogenetic research by ensuring that models and conclusions reflect the biological reality of the focal clade.

Attributes of the limbs, body shape, jaws, and even molecules have been used to investigate the role of diet and habitat adaptation in the diversification of South American Furnariidae birds (Claramunt et al., 2012), new world phyllostomid bats (Arbour et al., 2020; Rossoni et al., 2019), new world sigmodontine rodents (Maestri et al., 2017), Neotropical cichlids (Arbour & López‐Fernández, 2016; Astudillo‐Clavijo et al., 2015; Burress et al., 2018; Hauser et al., 2017; López‐Fernández et al., 2013), and South American characid fishes (Burns & Sidlauskas, 2019), among other mainland taxa (Huie et al., 2021). Such work has identified various clades that underwent early bursts and has elucidated the types of ecological niches that may have promoted diversification. This work has also revealed evolutionary patterns that are further suggestive of nested radiations, evolutionary constraints, and non‐adaptive accelerations in diversification on the mainland (Burns & Sidlauskas, 2019; López‐Fernández et al., 2013; Maestri et al., 2017). Taken together, accumulating natural history‐based studies indicate that, while ecological adaptation has indeed been an important source of diversity for some continental taxa, the relationship between ecological opportunity and both lineage and phenotypic diversification remains complex and in need of further study on the mainland.

## 4 NATURAL HISTORY AND ECOLOGY

Ecologists are broadly interested in understanding the fundamental rules that govern species interactions, from the individual to the ecosystem level. This frequently manifests in the form of experimental research, whereby variables of interest are systematically altered to observe their effects on the biological phenomenon of interest. For example, at the individual level, this may take the form of modifying ambient temperature to assess metabolic tolerance (Zhao et al., 2022). At the level of multispecies interactions, this may involve limiting resource availability among pre‐selected species pairs in order to determine the likelihood of competitive exclusion among conspecifics (Moldowan et al., 2015). Finally, at the community scale, this type of research may involve common garden experiments that seek to establish basic principles underpinning colonization and community assembly, which can then be extrapolated to the level of entire landscapes (Patterson et al., 2018). These methodologies are invaluable for a comprehensive understanding of how the natural world operates. However, we would argue that there are three principle limitations of experimental ecology that can be ameliorated by integrating natural history collections and observations: the non‐analogous and contracted timescales of controlled experiments, the difficulty in modeling the myriad factors that influence natural systems, and a lack of information that comes from primary observation necessary to inform those models.

The first limitation involves the temporal scope of experimental research. Rarely do ecological experiments, including well‐cited classic studies, exceed a few years in length (Condit et al., 1999; Findlay & Kasian, 1986; Simberloff & Wilson, 1969). Studies that rely on natural history observations, by contrast, can span much longer periods and therefore provide data uniquely suited to understanding natural biotic patterns, as well as their perturbations, at relevant timescales. Poignant examples include the multi‐year coral reef community structure dataset collected from the Buck Island Reef National Monument (Bythell et al., 2000); several multi‐decade‐spanning datasets being collected at the Algonquin Wildlife Research Station, including body size, nesting behavior, and nest temperature of turtles (Connoy et al., 2020), reproductive performance and survival of Canada jays (*Perisoreus canadensis*; Derbyshire et al., 2015), auditory monitoring of white‐throated sparrows (*Zonotrichia albicollis*; Falls, 1981), and population monitoring of small mammals (Fryxell et al., 1998); and, in an extreme and famous example, the 30‐year (at time of publishing) Darwin's finches dataset developed by Peter and Rosemary Grant (Grant & Grant, 2002). Data collected over such timespans are not only crucial for understanding how natural ecosystems function but are also the only way to test whether predictive models based on experimentation or simulation accurately reflect natural processes (Azaele et al., 2006). In essence, without an equivalent investment—of both effort and funding—into observational research, the applicability of laboratory and simulation‐based research to natural systems cannot be evaluated concretely.

Furthermore, many broader questions in the field of ecology cannot ever be assessed using neontological (modern) datasets. If the goal of ecology is to understand the relationships between species as well as their environments, then one of the principle goals of macroecology must be to understand these relationships over geological timescales. This is a goal that can only be achieved through an investigation of the fossil record, which represents the intersection of biology with the earth sciences. While the field of paleontology includes both experimental and simulation‐based paleontological research, the roots of the discipline are observational. The careful identification and description of tens of thousands of fossil organisms, the incorporation of geological and chemical data to reconstruct environmental conditions, and their painstaking stratigraphic correlations may not, by some definitions, represent experimental research. However, they form the basis for understanding life on this planet, which has developed over the course of billions of years.

With this type of quintessentially natural history‐based research, one can investigate how some of the earliest animal communities were structured (Finnegan et al., 2019) and how they changed over thousands to millions of years (Cullen & Evans, 2016; Nanglu et al., 2020). Observational research is at the heart of how biologists understand phenomena as fundamental as extirpation and extinction, including mass extinctions (Alroy et al., 2008) and the latitudinal diversity gradient (Jablonski et al., 2013). Poignant problems that face society today, such as increasing anthropogenic effects on species distributions, find their counterparts in the responses of ancient megafauna to both shifting climate change and human‐induced hunting (Gingerich, 2006; Wing et al., 2005).

The second limitation is the inherent difficulty in modeling the countless parameters of the natural world in a controlled environment. Part of the goal of experimental and simulation‐based research is to isolate the most powerful explanatory variables that govern a phenomenon of interest. As a result, any controlled work necessarily entails an abstraction from the natural system the researcher is concerned with. This is perfectly justified and, in fact, desirable in most circumstances. However, this only indicates that natural history and controlled experimental research should be working together in a feedback loop of self‐refinement. Natural history observations provide the foundation for hypothesis development; laboratory and computer simulations distill knowledge of natural phenomena into a set of codified “rules” for testing; and observational research allows for the ground‐truthing and testing of those rules and once again provides new data for robust methodological probing (Tosa et al., 2021). Observational research also permits the re‐examination of missing data and sources of analytical bias, providing insight into where knowledge is lacking and models or approaches may be underperforming (Benson et al., 2022; Brown et al., 2012, 2013; Nanglu & Cullen, 2023). Through these processes, taking the results of experimental research back into natural environments reintroduces the stochasticity and complexity of the true system, allowing for a full evaluation of the designed models. In many cases, the need to accurately model every parameter found in nature may be circumvented simply by returning to nature—an idea that seems obvious but is not always employed before sweeping claims are made regarding the generalizability of laboratory or computer‐based experiments. Ironically, but perhaps not surprisingly, many of the most influential early figures in ecology followed the philosophy of bringing theory back into a natural setting, whereas their academic descendants may not see the need for “basic” observational research and validation (Connell, 1961a, 1961b; Macarthur, 1958; Paine, 1974).

Finally, a focus on experimental and simulation‐based research programs to the exclusion of natural history reveals a mindset that assumes an unrealistically complete understanding of the natural world, highlighting a third limitation to experimental ecology. Undeniably, after detecting new ecological phenomena, whether it is a novel symbiosis, behavioral mode, or trophic strategy, laboratory work is essential in determining the relevance of observation‐based data to ecological theory more broadly. But the actual act of detection that drives hypothesis building is, by its own nature, an expression of observational science (Fellin & Schulte‐Hostedde, 2021; Millien et al., 2017). We hope that, by reading the examples presented above, readers do not believe we are trying to diminish the value of any one branch of ecological or evolutionary research. We are merely speaking in staunch defense of the exploration‐based work that provides the bedrock for robust research programs, allows researchers to address several fundamental questions in ecology and evolution, and often leads to breakthroughs that could not be anticipated in a lab.

### 4.1 Case study—Squeezing data from a stone: natural history museum specimens facilitate novel analyses of previously collected data

Natural history museums represent the primary repositories of specimens recorded from the natural environment over historical and deep‐time scales, including extant samples of complete individual organisms, tissues, molecular samples, fossils, minerals, and other geological samples. One important way in which natural history research can inform ecological, evolutionary, conservation, geological, and paleontological sciences comes from the potential use of specimens in novel consumptive analyses, which can provide critical insights that would otherwise be impossible. In many cases, museum specimens will have been collected during initial fieldwork surveys, inventory expeditions, or as part of a study with specific sampling objectives. The vast spatial, temporal, and taxonomic breadth that natural history collections encompass means that, as new destructive and non‐destructive sampling methods are developed, novel hypotheses will be testable, permitting the re‐use of these collections in ways well beyond the purpose initially envisioned for them.

At a basic level, access to various natural history specimens can facilitate parallel destructive research during the process of preparing specimens for permanent addition to a museum collection. Examples here include dissections to observe internal features as part of comparative anatomical studies or decay studies. The latter involves observing the natural decomposition of specimens under controlled conditions to inform understanding of taphonomy and its role in the preferential preservation of certain morphological characters, which can be of particular value in taxonomic and evolutionary studies of decayed or fossilized specimens (Murdock et al., 2014; Nanglu et al., 2015). Dissections can also complement more analytically complex approaches: some specimens may be dissected for high‐resolution imaging (e.g., via SEM, micro‐computed tomography [μCT], or similar approaches), often when whole specimens will not fit or the resolution depends on sample size, and thus imaging only the component of interest may maximize effectiveness. DNA and other molecular analyses are perhaps the most frequent consumptive use of museum specimens, wherein established collections act as a vast catalog of material for analysis and for comparison to newly collected field samples in ecological, phylogenetic, and/or evolutionary studies (de Carle et al., 2017; Hackett et al., 2008; Iwama et al., 2019; Langer et al., 2018). These collections also serve as the baseline from which DNA barcoding approaches can be developed and later verified (Kvist, 2013). Publicly available sequence data associated with museum specimens also allows researchers to validate previous taxonomic hypotheses in light of new data (Foote et al., 2022; Tessler et al., 2018).

Established museum collections, built over decades of consistent collecting, also facilitate the testing of hypotheses on temporal scales not otherwise possible for neontological studies. For example, a recent study of thousands of migratory North American bird specimens collected over nearly half a century found that, across dozens of species, overall body size has decreased and wing length has increased, likely in response to increases in global temperature (Weeks et al., 2020). This represents a quantitative and data‐rich example of biotic responses to climate change occurring in multiple vertebrate species over less than a single human lifespan—a result that would not have been obtainable without the presence of natural history museum collections and the many natural history studies that drove the building of those collections to draw upon. Put another way: much as the written word enables us to transfer complex information accurately across numerous generations, natural history collections provide us a record of specimens/data from earlier points in time for comparison with modern specimens and with modern approaches.

Paleontology is one field in particular in which the combination of new consumptive or destructive techniques with established natural history collection materials has enabled considerable recent advancements. In the last 30 years, vertebrate paleontology studies using osteohistological data from established museum specimens have revealed information and enabled testing of hypotheses related to the growth rates, ages at death, and physiologies of extinct and extant species (e.g., Botha & Chinsamy, 2001; Cullen et al., 2020, 2021; Erickson & Tumanova, 2000; Lee & O'Connor, 2013; Werning & Nesbitt, 2016); the developmental patterns of tooth implantation and attachment in various amniote groups (LeBlanc et al., 2017); and the understanding of life history data for use in future conservation programs (Marín‐Moratalla et al., 2013), to name just a few. This increasingly common methodology, which entails reconstructing life history information based on the internal microstructure of fossilized bones, reveals insights far beyond what is possible through examination of external morphology alone. Most of these osteohistology studies are based, at least partially, on museum specimens that were initially collected for use in natural history and taxonomic projects and were thus available for study as these new approaches were developed. Additionally, the combination of these existing historical collections with new sampling and consumptive biogeochemical methods has permitted detailed ecological information to be obtained and hypotheses of community and macroecology to be tested, including but not limited to: the differentiation of grazing versus browsing diets and habitat partitioning in extinct mammals (Bocherens et al., 1996; Koch, 1998; Zazzo et al., 2000); niche partitioning and testing for competition among co‐occurring large predators (Hassler et al., 2018); predator dietary shifts in response to environmental changes in European and Arctic settings before and after the last glacial maximum (Yeakel et al., 2013); community resource interchange and habitat use patterns among living vertebrates in coastal floodplain settings and their extinct counterparts in similar but still somewhat non‐analogous greenhouse climates (Cullen et al., 2019, 2020, 2022, 2023); and testing macroecological concepts such as the resource breadth hypothesis using stable isotope data from feathers obtained from birds in museum collections (Rader et al., 2017).

The intensive and geographically, temporally, and/or taxonomically broad sampling practiced in building natural history collections, often for the initial purpose of performing more descriptive/observational research characterizing biodiversity and taxonomy, can be loosely thought of as a form of scientific exaptation; previously collected materials are later re‐used in quantitative studies testing novel hypotheses using new methods that were not available at the time the specimens were initially collected. If these natural history collections did not exist and if the original groundwork of observational natural history research had not been performed, subsequent analyses addressing these more complex questions in ecology, evolution, and the earth sciences would not be possible. Not only are these later analyses also a form of natural history research, but they are, themselves, dependent on the foundation built by prior natural history collecting and study.

## 5 NATURAL HISTORY AND CONSERVATION BIOLOGY

Biodiversity on Earth is collapsing alarmingly quickly. If these trends continue unabated, the rate of species loss will be consistent with a sixth mass extinction (Barnosky et al., 2011). Through recorded history, the diversity of mammals—particularly nonhuman primates—has declined significantly (Ceballos & Ehrlich, 2002; Estrada et al., 2017); nearly one‐third of North American birds have disappeared (Rosenberg et al., 2019); and many amphibian species have rapidly gone extinct (Stuart et al., 2004). Most monitored invertebrate populations have faced mean declines of nearly half their historical abundances (Dirzo et al., 2014), with especially chilling biodiversity losses found in insects (see: Sánchez‐Bayo & Wyckhuys, 2019; Wagner et al., 2021). As a “mission‐oriented crisis discipline” (Soulé, 1986), the aims of conservation biology are to maintain, protect, and restore Earth's biodiversity through informed approaches that prevent the extinction of species due to human activity (Soulé, 1986). Ultimately, to establish effective conservation management plans, empirical data should be collected using the toolkit of natural history.

Natural history is essential to addressing the complexities of real‐world conservation problems. One such classic story is that of bowhead whale (*Balaena mysticetus*) censuses in Alaska during the 1970–1980s, when Iñupiat whalers were responsible for correcting erroneously low estimates of whale population size (Huntington, 2000). The traditional ecological knowledge gathered by the Iñupiat people over generations challenged government scientists' assumptions that bowheads could not be found beneath ice packs, leading to improved census methods that culminated in a *tripling* of the estimated population size (Huntington, 2000). Accurate estimations informed government management strategies for the species, allowing Iñupiat whalers to resume subsistence fishery activities without risk to bowhead conservation. Likewise, natural history knowledge of “legacy trees,” or old‐growth trees that escaped destructive human activities (Franklin, 1990), has led to their use in restoration efforts in abandoned cattle pastures, a conservation effort particularly salient in South America (Griscom & Ashton, 2011). The structural complexity provided by legacy trees invites a diverse array of fauna to the area (Mazurek & Zielinski, 2004), and the perching habitat they create acts as nucleation sites for seed dispersal (Verdu & Garcia‐Fayos, 1996). Ultimately, careful observation of these trees and their relationship to their ecosystem has cascaded into the extraordinary understanding that a single large tree can be a keystone for restoration initiatives.

Natural history also continues to play an important role in effective conservation management through long‐term monitoring, in spite of the ongoing devaluation of long‐term ecological studies (Tinkle, 1979). As just one example, long‐term monitoring of bald eagles correctly linked pesticide (DDT) use with reduced breeding success, which, in turn, led to an effective recovery strategy and reversed population declines (Grier, 1982). Similarly, long‐term monitoring of the Great Barrier Reef has led to key insights on the cumulative effects of different environmental stressors—including cyclones, rising sea temperatures, crown‐of‐thorns starfish, and river discharge—on coral reefs and informed management strategies (Thompson et al., 2021). Importantly, collections held within natural history institutions, including museums and herbaria, have played a critical role in guiding past and contemporary conservation research (Davis, 1996; Tewksbury et al., 2014). For example, natural history collections can provide a rich source of long‐term data that can be used to estimate both current and historical species distributions, ranges, and community composition, which can then be used to understand species' responses to anthropogenic stressors such as habitat loss (Pergams & Nyberg, 2001) and climate change (Dunn & Winkler, 1999; Hellberg et al., 2001; Parmesan et al., 1999; Weeks et al., 2020), as well as set informed benchmarks for the restoration of ecosystems (Hoeksema et al., 2011). Tissue collections held within many natural history museums have also been used for conservation genetics, for example, to determine genetic diversity baselines prior to environmental disturbance or the introduction of pathogens (Harper et al., 2006; Parker et al., 2011), and even to rediscover species that were once presumed to be extinct (Steeves et al., 2010). Natural history collections further serve as a teaching tool for taxonomy (Smith & Figueiredo, 2009), a discipline that is both essential for conservation and on the decline (Wheeler, 2007). Taken together, natural history and conservation biology are deeply intertwined. As environmental changes continue to impact ecosystems in dynamic ways, it is necessary to leverage natural history to create effective conservation strategies for the protection of species, ecosystems, and their services.

Furthermore, the lack of natural history knowledge can lead to failed conservation strategies that are harmful, costly, and hard to reverse. As an example, to aid in the recovery of kokanee salmon (*Oncorhynchus nerka*) populations in Okanagan Lake, Canada, opossum shrimp were intentionally introduced in 1996 as a potential food source, based on lab experiments showing that kokanee prey on opossum shrimp and early misinformed conclusions from their introduction at another lake (Welton, 2020). Once introduced, opossum shrimp avoided predation by remaining in deeper water during the day and shallow waters during the night, whereas kokanee showed the opposite pattern and instead became competitors with juvenile kokanee as they fed on their preferred zooplankton species during the night (Shepherd, 1999). With available food sources and no predator control, opossum shrimp populations grew significantly and continue to be a limiting factor for kokanee recovery (Andrusak et al., 2006; Vidmanic, 2008; Welton, 2020). As another example, in Chile, the development of Patagonia National Park accompanied well‐intentioned conservation strategies that required the cessation of native predator control and the removal of a non‐native domestic sheep population (Wittmer et al., 2013). However, a primary objective of Patagonia National Park was to protect Chile's national animal, the huemul deer (*Hippocamelus bisulcus*), and these abrupt changes led to large alternations in predator–prey dynamics that increased the predation of huemul deer by native predators, leading to eventual population declines (Wittmer et al., 2013). Here, utilizing natural history to understand the local ecosystem and the broader food web interactions held within could have prevented abrupt policy changes that led to further population declines of the endangered huemul deer. As a third example, forest fires were aggressively and systematically suppressed in the United States during the twentieth century, despite Indigenous knowledge that natural levels of forest fires are beneficial for forest management (Donovan et al., 2007). This misinformed strategy led to large‐scale ecological shifts, including changes in forest community composition, reductions in wildlife populations, and a buildup of forest fuel, which in turn contributed to more extreme fire patterns that now exceed $1 billion annually to control (Calkin et al., 2005; Donovan & Brown, 2007). As these examples demonstrate, conservation efforts require knowledge and appreciation of natural history to guide effective management plans.

### 5.1 Case study—Generations of observations: natural history as a lens for rare natural experiments

The Algonquin Wildlife Research Station (AWRS; Algonquin Park, Ontario, Canada) was founded in 1944 on the shores of Lake Sasajewun, a shallow body of water created in the early 20th century through damming of the Madawaska River. Since then, the AWRS has served as the home to a wealth of ecological studies on natural species, many of which continue to exist as long‐term studies that have spanned the careers of generations of researchers. One such study is the turtle life history project, which was initiated in 1972 by a team of researchers from numerous Canadian universities and continues to run to this day, a half‐century later.

The turtle project at the AWRS is, to our knowledge, the longest‐running continuous study of turtles in the world. Initial mark‐recapture studies of common snapping turtles (*Chelydra serpentina*) and eastern painted turtles (*Chrysemys picta*) on the Sasajewun Dam conducted in the 1970s (Loncke & Obbard, 1977) would eventually burgeon into projects in numerous disciplines, including behavior (Keevil et al., 2017; Moldowan et al., 2020; Obbard & Brooks, 1979), demography and life history (Galbraith et al., 1989; Galbraith & Brooks, 1987; Samson, 2003), developmental physiology (Massey, Congdon, et al., 2019; Massey, Holt, et al., 2019; Rollinson et al., 2018; Rouleau et al., 2019), feeding ecology (Moldowan et al., 2015), methods in conservation (Riley & Litzgus, 2013), genetics (Rouleau, 2020), and reproductive ecology (Obbard & Brooks, 1980, 1987); by no means is this list exhaustive. Of these, one of the most incredible testaments to natural history's role in informing conservation is the story of a rare natural experiment that occurred in the late 1980s.

During the winters of 1986–1989, the study population of snapping turtles at the AWRS faced a series of mortality events in which predation by otters (*Lutra canadensis*) decimated approximately half of the hibernating adults. At the time, little was known about snapping turtles, nor indeed, the knowledge requirements for conservation of long‐lived species in general (Brooks et al., 1988; Dodd, 1985). Informed by over a decade of observations on adult survivorship and early‐life recruitment of snapping turtles, Dr. Ronald J. Brooks and colleagues made the astute prediction that the slow life history of snapping turtles would result in extremely slow population recovery (Brooks et al., 1991). In light of other destructive activities such as hunting and habitat fragmentation, they were also able to hypothesize the risk that stochastic mortality events presented to the conservation of snapping turtles and other long‐lived species (Brooks et al., 1991).

Nearly three decades after these mortality events were observed, the population recovery of AWRS snapping turtles was revisited (Keevil et al., 2018). New data from the long‐term turtle project described a limited signal of population recovery *even after* enough time had passed to establish a new generation of adult snapping turtles (Keevil et al., 2018). Moreover, population recovery was absent despite both extremely high adult survivorship and connectivity with other snapping turtle populations, factors that would otherwise be expected to replenish abundance (Keevil et al., 2018). Ultimately, continued monitoring of this population allowed researchers to record the empirical results of this natural experiment and, later, confirm the post‐catastrophe predictions made decades earlier.

For the purposes of the conservation of snapping turtles and other long‐lived species, several conclusions were established based on the snapping turtle mortality event and the long‐term monitoring program more generally. First, stochastic and acute catastrophic events can have devastating impacts on populations. In the present case, it took at most, a few individual predators to decimate an entire population that typically experiences extremely high adult survivorship. Although the frequency and intensity of such events cannot be predicted, their risk should be appreciated, especially under management frameworks. Next, for long‐lived creatures with slow life histories, recovery can occur on very large timescales—so long that they may span numerous generations of researchers themselves. Heeding this fact is crucial in populations with low recruitment of young into the adult pool. It thus follows that the importance of protecting reproductively mature adults must be underscored in the conservation of organisms with slow life histories. Many of these ideas have been adopted into management frameworks for snapping turtles and other chelonians (COSEWIC, 2008), serving as theoretical justifications to promote the persistence of at risk, long‐lived organisms through numerous ongoing efforts (e.g., ecopassages, Read & Thompson, 2021); improved road planning (Langen et al., 2012); and protection from hunting and persecution (Environment and Climate Change Canada, 2020). Importantly, the two studies (Brooks et al., 1991; Keevil et al., 2018) describing this tragic natural experiment offer a lesson on the importance of continuing to make observations of the natural world. In the absence of the long‐term study, the decline of the population would still have occurred, but no one would be any wiser.

## 6 NATURAL HISTORY AND BROADER IMPACTS

### 6.1 Natural history education contributes to a scientifically literate society

In North America and worldwide, there have been declines in public engagement with nature (Pergams & Zaradic, 2008), driven by factors including urbanization, a lack of exposure to nature, increasing time spent with technology, and influences related to socioeconomic status (Louv, 2008). Because natural history involves the study of one's surroundings, learning can occur at an individual level, from tangible objects to observable phenomena, without formal training or specialized equipment. This high degree of accessibility makes natural history particularly approachable within the natural sciences. The ease of entry into natural history allows individuals to make personal connections and apply new understandings to their immediate surroundings, promoting curiosity, self‐driven inquiry, and a greater quality of life (Kashdan & Steger, 2007; National Research Council, 2009; Simon, 2001).

We are constantly reminded, in our collective experiences as educators and museum professionals, that students and members of the general public, children and adults alike, show an innate curiosity for science and natural history. This curiosity is particularly evident when one is engaged in experiential learning—whether that is showing specimens from museums or out in nature, or relating natural history to other parts of life (e.g., how components in makeup, technology, and food are sourced)—but is also observable in the continued popularity of science media (e.g., nature documentaries) and, to some degree, pseudoscience (e.g., astrology, healing crystals).

Higher levels of education, specifically scientific education, are correlated with greater trust in the scientific method and lower susceptibility to misinformation (Funk et al., 2019; National Science Board & National Science Foundation, 2020; Roozenbeek et al., 2020). Natural history institutions, such as museums, zoos, botanical gardens, and aquaria, play a vital role in engaging a wide audience with science outside of, or in conjunction with, formal education (Mujtaba et al., 2018; National Research Council, 2009; Schwan et al., 2014). They can accomplish this in many ways, including but not limited to: displaying physical exhibitions and interpretive text; developing popular tools for natural history enthusiasts (e.g., California Academy of Science's iNaturalist, Cornell Lab of Ornithology's eBird); and facilitating a variety of public programming such as family‐oriented events (e.g., Family Day at the Smithsonian Institution National Museum of Natural History, Open House at the Canadian Museum of Nature); social media engagement (e.g., the Twitter accounts of Monterey Bay Aquarium and the Field Museum's Sue the *T*. *rex*); and community science initiatives such as biodiversity surveys (e.g., BioBlitz; Peter et al., 2021). We frequently find that, at multidisciplinary outreach events, the public is more interested in learning about other natural history subjects than the discipline that initially drew them to participate, expanding their breadth of knowledge in science. Our personal experiences are reinforced by data showing that informal science experiences such as these support personal and social development, particularly for disenfranchised individuals (Wellcome Trust, 2019).

Many of the most pressing global issues are deeply intertwined with the scientific process and the natural world. Rising global temperatures, pollution, antibiotic resistance, extinction, habitat loss, unsustainable resource extraction, and the emergence of new diseases—among other perils—continue practically unabated (e.g., IPBES, 2019; Jones et al., 2008; NOAA, 2022; Shaddick et al., 2020; UNEP, 2019; WHO, 2015). At the same time, misinformation, pseudoscience, political and consumerist propaganda, and social media are contributing to vaccine hesitancy, anxiety about nuclear power and genetically modified organisms, and skepticism about climate change and the COVID‐19 pandemic (Funk et al., 2019; Larson, 2018; OECD, 2020; Rainie et al., 2015; Wellcome Trust, 2018; WHO, 2021). Although acceptance of anthropogenic climate change and the reality of COVID‐19 have increased (Fagan & Huang, 2019; Funk & Tyson, 2021), skepticism surrounding these crises persists, and the rise of populism has ushered in growing polarization over the magnitude of the issues and how to address them (Chinn et al., 2020; Funk & Kennedy, 2020; Funk & Tyson, 2021; Larson, 2018; Masoud et al., 2021). Some of these issues require scientifically informed solutions, whereas others stem directly from discomfort or distrust surrounding science. Critically, viewpoints stemming from an understanding of science (or lack thereof) directly impact public policy for generations to come through a variety of avenues, including local initiatives, voter‐approved measures, and government representation. In addition to creating a more scientifically literate society, the work of natural history institutions can also inspire the public, including politicians, educators, and industry leaders, who will help shape our collective future.

### 6.2 Natural history education improves inclusivity, diversity, and equity in access to science education and exploration

Natural history holds tremendous potential for inclusive scientific outreach that targets historically underrepresented communities, including Black, Indigenous, and People of Color (BIPOC), members of the LGBTQ2S+ community, people who have disabilities, and people from low‐income backgrounds. Beyond moral obligations in pursuit of equity, increasing engagement with underrepresented communities is also of particular importance, as many of the threats facing the natural world and society, including environmental and climate crises, continue to disproportionately impact marginalized communities (Cutter & Finch, 2008; Ford, 2009; IPCC, 2014).

Although Western natural history practices have, and continue to be, exclusionary for reasons including European modes of knowledge production and translation (Radcliffe, 2017) and inequitable access to natural spaces (Cutter, 1995) and natural history institutions (Das & Miranda, 2018), growing awareness of these issues are leading to positive and equitable changes within the discipline. For instance, long‐standing Indigenous natural paradigms are now being incorporated into Western research, management, and ecology education, leading to positive impacts in both knowledge generation and student training (Bartlett et al., 2012; Mantyka‐Pringle et al., 2017). Indeed, the field of natural history is uniquely positioned for cross‐cultural enhancement through conceptual frameworks such as Etuaptmumk (“Two‐Eyed Seeing”), because of shared philosophical foundations including human–environment interconnectedness, appreciation for experiences in nature, and preservation of land and water (McKeon, 2012). Inclusion of diverse perspectives in natural history can further serve to increase the recruitment and retention of historically underrepresented students by dismantling dominant stereotypes of science culture, thus improving students' sense of belonging in their ecology programs (O'Brien et al., 2020; Puritty et al., 2017).

Nature education, more generally, may also serve as a particularly effective springboard for students' subsequent STEM education, especially considering that a lack of comfort and experience outdoors is a major barrier to the recruitment of historically underrepresented scholars in STEM (O'Brien et al., 2020). Nature outreach can be performed locally within communities, connecting students' lived experiences to their learning—a core principle of authentic learning strategies (Herrington, 2005). Education in natural settings also improves students' engagement and comfort in learning environments, promotes pro‐environmental behaviors in students, and improves outcomes for students who are underserved by traditional classroom teaching (Kuo et al., 2019). Further, natural history outreach may present fewer barriers to both students and educators, given that many exploratory activities in nature or natural history institutions may require little to no equipment, fees, or lesson planning.

Already, several grassroots initiatives have leveraged natural history to promote equitable access to science, whereby historically underrepresented students explore and connect their values and sense of self to the natural places they are immersed in (e.g., Riparia, Black Birders Week). As one such example, Diversity of Nature, a BIPOC‐focused outreach organization, takes students to natural sites where they learn hands‐on and diverse scientific content (e.g., Indigenous ecology, which is rooted in observation‐based natural history) from BIPOC naturalists (Massey & Arif, 2021). These experiences can provide inclusive and targeted opportunities for underrepresented youth to cultivate their love of science while simultaneously providing opportunities for diverse naturalists to give back to their communities, an important desire for many historically underrepresented scholars (Massey et al., 2021; Smith et al., 2014; Thoman et al., 2015).

Given the positive benefits of natural history in instilling curiosity, science literacy, greater academic success, and long‐term interest in science (Beltran et al., 2020 ;Mujtaba et al., 2018), natural history institutions, including museums and science centers, and those who work within them, should continually strive to create inclusive programming, exhibits, and training to increase engagement with historically underrepresented communities. This is particularly important, as these institutions act as authorities on nature for their audiences, offering a public image of the natural sciences and the way research is conducted across these disciplines (Cassidy et al., 2016). For example, natural history institutions can create accessible programming (Pressman & Schulz, 2021), highlight diverse scientific perspectives (Lee et al., 2020), facilitate inclusive pedagogical training (Tam et al., 2000), and continually work toward decolonizing collections and teachings (Das & Miranda, 2018; Onciul, 2015; Turner, 2014; see Box 1). Diversity enhances the quality, quantity, and impact of science (Freeman & Huang, 2014; Hofstra et al., 2020; Hong & Page, 2004); making room for diverse perspectives, scientists, and community members within natural history will similarly enhance the discipline (AlShebli et al., 2018; Murphy et al., 2020; Swartz et al., 2019). Ultimately, greater inclusivity within the natural history discipline will benefit underrepresented communities, broader society, and our collective understanding of natural history and the sciences.

## 7 NATURAL HISTORY FOR THE FUTURE

The discussions and case studies above lay out a case for the importance of natural history knowledge—now and historically—and advocate for the continued funding of natural history research and programming (Figure 2). To that end, we include a list of recommendations and actionable items that we believe will help maintain the health of natural history science as an integral part of any scientific discipline (Box 1). These recommendations come from our personal experiences as researchers and educators, particularly given our significant collective experience with natural history research and collections. An important point we have yet to address is that a complete understanding of the ways in which natural history is important to current and future research is utterly unattainable. A vexing and delightful aspect of this field of research is that, by definition, it is impossible to anticipate every observation and phenomenon, let alone the ways in which these data may be relevant or essential in the future. To illustrate this need for fundamental, exploratory science, many have used the analogy of searching for gold only in places where it has already been found.

Consider that new uses of natural history collections and observations are finding new life daily through novel forms of analyses that their collectors could not have imagined. A series of recent studies have highlighted this phenomenon. Entomological (Grewe et al., 2021) and mammalian (Roycroft et al., 2021) specimens, some of which were collected nearly 200 years ago, suddenly find themselves critically important in the era of conservation genomics. The emergence of new and better sequencing technologies, coupled with an ever‐increasing body of genomic resources, allows researchers to wrest more and more genetic and epigenetic information from formalin preserved or otherwise degraded specimens (Hahn et al., 2021; Straube et al., 2021). Ichthyological collections hold new promise for understanding changes in parasite communities (Fiorenza et al., 2020; Welicky, Preisser, et al., 2021), changes in trophic level (Welicky, Rolfe, et al., 2021), and microplastic uptake (Hou et al., 2021) over multi‐decadal timescales. Other similar and novel approaches can be pursued using museum wet specimens, such as examining stomach contents and utilizing them to infer dietary data in macroevolutionary and functional ecological analyses (Arbour et al., 2020), sequencing bacterial endosymbionts to study their function and the history of their host associations (Manglicmot et al., 2020), or sequencing the gut contents of leeches as a tool for monitoring mammalian biodiversity in tropical settings (Weiskopf et al., 2018), an approach that could yield important insights into historical biodiversity estimates and the examination of temporal changes in biodiversity. Paleontological collections similarly contain massive amounts of currently unrecognized data that are becoming available with the advent of new technologies. The isotopic analyses we describe in the “Natural history and ecology case study” above (Cullen et al., 2020), as well as the increasingly common recovery of ancient DNA (Smith et al., 2021; van der Valk et al., 2021), are both examples of extractive analytical methods beyond the original intention of most fossil collections. New, non‐extractive imaging methods—such as synchrotron imagery, μCT, and neutron imaging—are also generating exciting new structural, morphological, and life history data from biological and geological specimens that can seem outwardly unremarkable (Moon et al., 2022; Rakovan et al., 2019; Zhai et al., 2019).

This is to say nothing of the many studies that rely on data that was originally collected for primarily observation or curiosity‐driven reasons and have subsequently demonstrated value to other studies down the line. The tens of thousands of birds used by Weeks et al. (2020) to demonstrate how warming temperatures have consistently resulted in body size reduction in 52 species were opportunistically scavenged by both professionals and volunteers from the Field Museum in Chicago over decades. Diamond specimens with fluid inclusions were eventually used to unveil the presence of aqueous pockets in the transition zone beneath the Earth's upper mantle (Tschauner et al., 2018). In the words of the authors themselves, the most comprehensive study of parasite extinction risk, forecasting a 5%–10% loss of diversity by 2070, “would be essentially impossible without taking advantage of existing data infrastructure, especially from natural history collections” (Carlson et al., 2017). These developments are just some examples of how data that already exists within natural history repositories are untapped wellsprings to test new hypotheses.

These new forms of data likely represent only a small fraction of what is housed in natural history collections, with the vast majority of specimens not receiving adequate maintenance, intensive study, publication, or deposition into publicly available databases (Escobar, 2018; Marshall et al., 2018). Combine this lack of attention with the fact that only a small fraction of the world's total animal and plant diversity has been sampled (Higgs & Attrill, 2015; Hughes et al., 2021; Stork, 2018), to say nothing of fungi, bacteria, and other microorganisms, and a clear picture begins to emerge: a picture in which the loss of natural historians, taxonomists, and observational research programs represents the divorce of the scientific method from its original *raison d'être*—to understand, as wholly as possible, the natural world in all its diversity and complexity.

## 8 CONCLUSIONS

1. Natural history research is crucial to every scientific discipline, particularly the biological and earth sciences. The data this research generates underpins the creation of informed hypotheses. In turn, examining the applicability of models and theories to natural systems is a fundamental goal of these disciplines.
2. Natural history research is indispensable for creating a scientifically literate society and informing policy decisions. This is particularly true in an era when issues of biology and conservation (i.e., pandemics, climate change, and habitat destruction) are at the forefront of current global crises.
3. Far from being a relic of the past, natural history research often incorporates the most modern methods available, and is a continuously evolving scientific discipline.
4. It is impossible to foresee all the future uses of collections, making the need to protect them even more urgent. Each specimen is unique, representing an irreplaceable part of the Earth's history. Therefore, the loss of specimens (or worse, entire collections) represents both an immediate loss and the loss of unknown potential value.

BOX 1 **Principles and guidelines for supporting natural history research.**

To synthesize the insights from this manuscript, we have assembled a series of actionable items for supporting natural history research and integrating them into your own education, research, and institutions. We specifically gear these items toward three groups: (1) current or prospective undergraduate students who are interested in becoming involved with natural history research, or who may wish to learn more about natural history resources and career paths available to them; (2) researchers, including graduate students, who are currently pursuing natural history research, or who may be interested in integrating natural history data and methods into their research; and (3) administrators, who often shoulder the responsibility of determining which programs are highlighted, supported, and funded. There are many ways to integrate natural history data into your institutions and research programs. Ideally, these approaches will be taken holistically and at all levels, from student education to hiring decisions to the distribution of support and funding.

For students

1. **Get involved with natural history experiences in your community.** Many community groups and institutions can open doors to valuable natural history research and collection experiences led by skilled mentors. These may include local naturalist clubs, stewardship programs, arboreta, botanical gardens, and museums. Citizen science events and initiatives—such as bioblitzes—often provide valuable opportunities to work alongside, and learn from, experienced natural historians. You can also leverage modern tools, such as iNaturalist—we recommend the “Seek” application—and eBird to connect with a community of naturalists online. Data collected by citizen science programs such as these have the added benefit of supporting ongoing research programs both locally and internationally.
2. **Incorporate natural history into your formal education.** If you are a current undergraduate student, look for field courses and taxon‐specific diversity courses (e.g., entomology, mycology, botany, ichthyology, paleontology, etc.) within your institution. There may also be courses that do not have natural history as their primary focus but that include field or specimen‐based experiences as labs, tutorials, or field trips. Keep in mind that you do not necessarily need to enroll in a biology department to take these courses. Natural history is a great way to build skills that are applicable to many fields, both within and outside of STEM (see point 4).
3. **Seek out natural history research experience at your university.** If you are considering a career in natural history (be it in the research, museum, education, government, or public sectors), conducting research at the undergraduate level is one of the most valuable things you can do. In addition to giving you a taste for the practice of natural history research in an institutional setting, these experiences will help you build vital analytical skills (e.g., programming, statistics) and are valuable additions to your CV, especially if you choose to pursue a post‐secondary degree. Look for research opportunity programs that help fulfill your degree requirements, as well as paid opportunities such as work study programs and research grants, which are often available from government organizations.
4. **Cultivate transferable skills from your natural history work and interests.** Many of the skills developed by those who take an early interest in natural history activities (e.g., bird watching, rock collecting, tidepooling) are not only critical for any scientist but also applicable to many aspects of life in general. For burgeoning scientists, these activities provide opportunities to participate in field work, maintain rigorous field notes, and build familiarity with natural systems that will help to contextualize theoretical knowledge. Crucially, identifying specimens explicitly involves formulating and testing hypotheses. (e.g., “Based on the shape of the antennae, I think this is a scarab beetle. Are there other features that support or refute this identification?”). Other skills—such as recording, organizing, and aggregating data; recognizing patterns and trends; and differentiating important information from noise—are valuable in any career setting.
5. **Reach out to researchers whose natural history work you admire.** Often, they will be excited to share their interests with you and may be able to point you toward natural history opportunities or provide helpful advice about project ideas, grant applications, graduate school, and other scientific endeavors. Do not forget that many of these researchers (including the authors), started out in very much the same way you are now.
6. **Stay curious about the natural world.** Scientific breakthroughs can come from a chance observation as readily as reading papers (although that's certainly valuable too). Remember, if natural history is what got you interested in science in the first place, abandoning it in pursuit of something that seems more scientifically *en vogue* is doing yourself a disservice. Instead, find a way to integrate natural history with your developing interests.

For researchers

7 **Combine natural history with theoretical and lab‐based research programs.** Encourage the acquisition of primary data where appropriate, and acknowledge the natural historical basis of your research at talks, in grants, and to prospective students. Whether your research is primarily theoretical or lab‐based, consider pursuing opportunities (e.g., student‐led projects or collaborations) to validate or contextualize your findings in natural systems.
8 **Emphasize the utility of natural history collections in understanding complex topics in ecology and evolution.** When these collections are not easily accessible (i.e., museum access may not be logistically or financially feasible), consider making use of freely available online resources such as online collections like those offered by The Hunterian (https://www.gla.ac.uk/hunterian/collections/searchourcollections/) and the Royal Ontario Museum (https://www.rom.on.ca/en/collections‐research/online‐collections) and the educational resources offered by the University of California (https://evolution.berkeley.edu/; https://undsci.berkeley.edu/) or the Smithsonian National Museum of Natural History (https://naturalhistory.si.edu/education/natural‐history‐summer‐explorations/paleo‐art‐edge‐extinction).
9 **Support long‐term studies and monitoring programs.** The data derived from these programs are invaluable for tracking change through time and establishing the baselines against which phenomena such as climate change, trait evolution, and dispersal patterns are measured. The collection of these data is also time‐sensitive; each natural observation is a unique data point that cannot be perfectly replicated. Wherever possible, consider using these long‐term datasets in your own research and teaching, or supporting these initiatives in other ways (e.g., by contributing observations or assisting with the administrative work required to maintain them). Ultimately, establishing a long‐term collaboration with these types of programs, or creating one yourself, may be the best way to ensure the continued health of natural history research.
10 **Foster collaborations that incorporate natural history knowledge.** Integrating data from natural history research into broader meta‐analyses or modeling allows the strengths of each approach to be complementary while mitigating potential sources of uncertainty. As natural history researchers, we are eager to use the specialist knowledge of our systems to contribute to the broad questions other research groups are asking. Also, look to include insights from Indigenous Knowledge Holders and Knowledge Systems wherever possible and to partner with Indigenous and local communities where appropriate (Wong et al., 2020).
11 **Cultivate natural history skills in your students.** The skills implicit in many natural history research methods are applicable to diverse aspects of science and life in general (see point 4). Taking and maintaining good field notes is a particularly valuable skill. This means attaching relevant metadata to observations and specimens. What constitutes relevant metadata varies widely depending on your system, but should minimally include: GPS coordinates, time of day that the observation was taken, ambient environmental conditions, and associated fauna and flora. In addition to written observations, field notes may also constitute other sources of information, such as drawings, photographs, or pressed botanical specimens. Note‐taking is critical in any discipline, but particularly in natural history studies, where that observation may not be made again (see point 8).
12 **Deposit your data into museums and public databases whenever possible.** For natural history research to keep pace, data need to be accessible and well documented so that other researchers can contribute to projects beyond the scope of their original purpose. This is also essential for ensuring that research is reproducible. When writing budgets or applying for grants, consider including funding to support the deposition and curation of specimens in natural history collections.
13 **Share your natural history knowledge and research with the public through outreach events or social media.** This can also help extend the reach of your papers to new and broader audiences.

For department heads and administrators

14 **Support and encourage natural history research within your institutions and departments.** Establish funds within your institutions to financially support natural history work, which can include museum visits, fieldwork, field station infrastructure, and costs of analyses and equipment. Highlight the contributions of natural history researchers (e.g., through departmental awards and recognition), and ensure that natural history research is a visible component of seminar series and events designed to showcase departmental research. Continue to financially support such structures that already exist. Although natural history programs may be expensive, consider that they often have disproportionately large positive effects on research and education, both within an institution and more broadly (see section 6). Fundamentally, these programs provide data and information crucial to the formation of novel hypotheses that are the linchpins of many science departments.
15 **Seek out (and hire) researchers with natural history experience and expertise.** The next generation of principal investigators is determined by search committees, which often operate out of the sight of the general scientific community. In essence, you are the ones that determine the health of the natural sciences through the value judgments that are made in selecting new faculty.
16 **Make natural history a required component of undergraduate curricula.** Courses in which students gain first‐hand experience with natural history research skills (e.g., specimen collection, identification, preparation, dissection, maintaining a field notebook) foster the development of observational skills and demonstrate the value of taxonomic expertise. While some universities already offer courses fitting this description—field courses and taxon‐specific diversity courses—they are frequently optional. As a result, students may forgo these courses in favor of those that are required or that seem more academically “important” and “rigorous.” Furthermore, field courses are often prohibitively expensive for many students and often occur during time periods that interfere with employment, like the summer months. We suggest incorporating natural history courses into undergraduate degree requirements and offering more accessible field experiences. This can be achieved by including field experiences in existing courses, offering field courses that are closer to home (e.g., local parks and conservation areas), or subsidizing field courses for lower‐income students. Resources offering example curricula, guidelines, and outcomes, such as the AAAS Vision and Change report on undergraduate biology education (Brewer & Smith, 2009), can provide useful guidance on this and other action items.
17 **Encourage natural history‐focused public engagement.** Outreach is a vital method of building public excitement and confidence in science, and—as we have demonstrated—natural history is particularly well‐suited for this purpose. Natural historians within your institution should be encouraged to engage with the public and local communities, and their efforts should be explicitly valued as acts of service to the department.
18 **Acknowledge that a quality STEM education is not equally accessible to everyone.** Racial, social, economic, and other factors contribute to demographic biases in academic communities. Natural history research, as an approachable access point for science education, can help broaden perceptions of what scientists look like, and contribute to more equitable and accessible participation in science.

## AUTHOR CONTRIBUTIONS

**Karma Nanglu:** Conceptualization (lead); visualization (supporting); writing – original draft (equal); writing – review and editing (equal). **Danielle de Carle:** Conceptualization (equal); visualization (supporting); writing – original draft (equal); writing – review and editing (equal). **Thomas M. Cullen:** Conceptualization (equal); visualization (supporting); writing – original draft (equal); writing – review and editing (equal). **Erika B. Anderson:** Conceptualization (equal); writing – original draft (equal); writing – review and editing (equal). **Suchinta Arif:** Conceptualization (equal); writing – original draft (equal); writing – review and editing (equal). **Rowshyra A. Castañeda:** Conceptualization (equal); writing – original draft (equal); writing – review and editing (equal). **Lucy M. Chang:** Conceptualization (equal); writing – original draft (equal); writing – review and editing (equal). **Rafael Eiji Iwama:** Writing – original draft (equal); writing – review and editing (equal). **Erica Fellin:** Conceptualization (equal); writing – original draft (equal); writing – review and editing (equal). **Regine Claire Manglicmot:** Conceptualization (equal); visualization (lead); writing – original draft (equal); writing – review and editing (equal). **Melanie D. Massey:** Conceptualization (equal); writing – original draft (equal); writing – review and editing (equal). **Viviana Astudillo‐Clavijo:** Conceptualization (equal); writing – original draft (equal); writing – review and editing (equal).

## CONFLICT OF INTEREST STATEMENT

The authors declare no competing interests.

## Data Availability

No new data were generated for this review.
